# Clinical Outcomes of Radial Collateral Ligament Repair in Recalcitrant Lateral Epicondylitis with Mild Elbow Instability Following Repeated Steroid Injections

**DOI:** 10.3390/jcm14186474

**Published:** 2025-09-14

**Authors:** Sang Won Moon, Darryl D’Lima, Jin-Young Bang

**Affiliations:** 1 Department of Orthopedic Surgery, Inje University College of Medicine, Haeundae Paik Hospital, Busan 48108, Republic of Korea; 2 Shiley Center for Orthopaedic Research and Education at Scripps Clinic, Department of Molecular Medicine, Scripps Research, La Jolla, CA 92037, USA

**Keywords:** lateral epicondylitis, radial collateral ligament, steroid injection, elbow instability, ligament repair

## Abstract

**Background:** Lateral epicondylitis is a common degenerative condition of the elbow, often managed conservatively. However, a subset of patients who had recalcitrant symptoms and repeated corticosteroid injections may develop subtle lateral elbow instability, particularly involving the radial collateral ligament (RCL). This study aimed to evaluate the clinical outcomes of RCL repair combined with open ECRB debridement in patients with chronic lateral epicondylitis and mild instability. **Methods:** We retrospectively reviewed 92 patients who underwent surgery for recalcitrant lateral epicondylitis between 2016 and 2022. Twelve patients with imaging and intraoperative findings of mild lateral instability underwent open RCL repair with or without LUCL augmentation (unstable group). The remaining 80 patients without instability underwent arthroscopic ECRB release and drilling (stable group). Pre- and postoperative outcomes were assessed using VAS, MEPS, DASH, and range of motion. Comparative analyses were performed between the two groups. **Results:** All unstable patients had a history of repeated steroid injections (mean 3.4) for 18 months. Postoperatively, pain scores (VAS) improved from 6.8 to 1.4, MEPS increased from 53 to 91, and DASH decreased from 47.1 to 13.8. No major complications or recurrent instability were observed at one-year follow-up. Subgroup analysis revealed that older age, previous surgery, and a greater number of injections were significantly associated with instability. **Conclusions:** RCL repair combined with ECRB debridement yields favorable clinical outcomes in patients with recalcitrant lateral epicondylitis and mild instability, especially those who had a history of repeated corticosteroid injections. Proper evaluation and surgical repair of underlying ligament pathology may improve outcomes in this subset of patients. Favorable clinical outcomes were defined as improvements compared to preoperative baseline scores. These outcomes were compared to preoperative scores and exceeded MCID thresholds where applicable.

## 1. Introduction

Lateral epicondylitis, commonly known as tennis elbow, is a frequent cause of lateral elbow pain that arises due to degenerative changes and microtears in the origin of the common extensor tendon, particularly the extensor carpi radialis brevis (ECRB) [1,2]. Most cases respond to conservative treatment including rest, physical therapy, counterforce bracing, and corticosteroid injections. However, a subset of patients—estimated at 10–20%—fail to improve with conservative measures and may require surgical intervention such as ECRB debridement [3]. Corticosteroid injections, while effective for short-term pain relief, have been associated with detrimental long-term effects, particularly in patients who receive repeated injections. Multiple steroid injections can exacerbate tendon degeneration and may increase the risk of partial tendon rupture and adjacent ligament injury [4,5,6]. Despite the known limitations of steroid use, their long-term implications on elbow stability—especially involving the lateral ligament complex—remain under-recognized. The lateral ligament complex of the elbow comprises the radial collateral ligament (RCL), lateral ulnar collateral ligament (LUCL), and annular ligament [7]. Although the lateral ulnar collateral ligament (LUCL) is traditionally considered the most important stabilizer against elbow instability, the significance of the radial collateral ligament (RCL) has also been increasingly recognized [8,9,10,11]. The recent literature suggests that unrecognized RCL injury may contribute to symptomatic instability in recalcitrant lateral epicondylitis, particularly in patients with a history of multiple steroid injections [12].

However, to date, there is a lack of focused clinical data evaluating the outcomes of RCL repair in patients with refractory lateral epicondylitis who present with mild instability. Furthermore, the interaction between steroid-induced tendon degeneration and subtle elbow instability remains poorly characterized. Given this gap, we aimed to investigate the clinical outcomes of open debridement combined with RCL repair, with or without LUCL augmentation, in patients with recalcitrant lateral epicondylitis and suspected mild instability.

We hypothesized that patients who had a history of repeated steroid injections are more likely to present with subtle RCL injury, and that targeted repair of this ligament, when indicated, would improve pain and functional outcomes.

### 1.1. Study Design and Patient Selection

This retrospective case series was performed at a single tertiary academic medical center. Institutional Review Board (IRB) approval was obtained prior to data collection. The medical records of patients who underwent surgical treatment for recalcitrant lateral epicondylitis between January 2016 and December 2022 were reviewed. Recalcitrant lateral epicondylitis was defined as persistent lateral elbow pain lasting more than 12 months despite comprehensive conservative treatment, including rest, physical therapy, non-steroidal anti-inflammatory drugs (NSAIDs), counterforce bracing, and corticosteroid injections.

Inclusion criteria for recalcitrant lateral epicondylitis were:

- Age between 30 and 70 years.
- Clinical and radiologic diagnosis of chronic lateral epicondylitis.
- Failure of ≥1 year of conservative management.
- Availability of complete medical records and ≥12 months follow-up.

Exclusion criteria for recalcitrant lateral epicondylitis were:

- Prior fracture or dislocation around the elbow.
- Systemic inflammatory diseases (e.g., rheumatoid arthritis).
- Full-thickness tears of the common extensor origin on MRI.
- Workers’ compensation or ongoing legal disputes related to the elbow.

**Figure 1:**
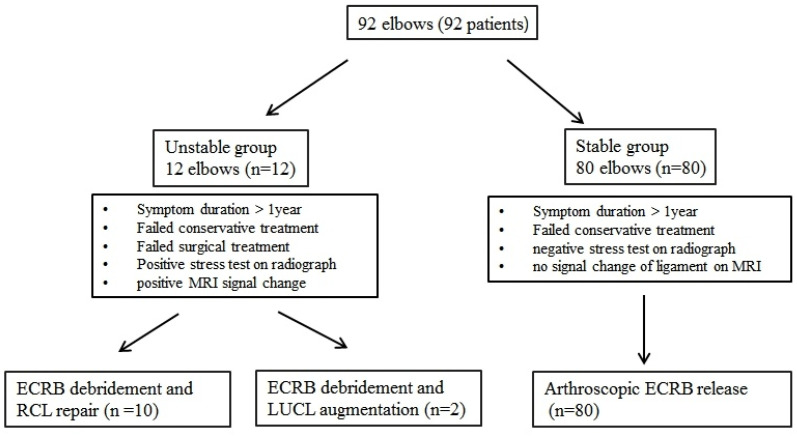
The flowchart for the patient selection and clinical process. ECRB, extensor carpi radialis brevis.

Of the 92 eligible patients, 12 demonstrated clinical and radiological signs of mild elbow instability and underwent RCL repair with or without LUCL augmentation. These 12 cases constituted the unstable group. The remaining 80 patients, without instability, were designated as the stable control group for comparative purposes (Figure 1).

### 1.2. Evaluation of Instability

Chronic lateral epicondylitis diagnosis was based on persistent lateral elbow pain for >6 months, localized tenderness over the lateral epicondyle, and MRI confirmation of extensor tendon pathology. All patients underwent standardized varus stress testing on radiographs as part of our institutional protocol.

In this study, mild lateral instability was defined as a capitellum–sublime tubercle–radial head angle (CSRA) exceeding 5°, confirmed by both physical examination and intraoperative stress testing, in the absence of a positive provocation test typically seen in cases of complete LUCL tear.

**Figure 2:**
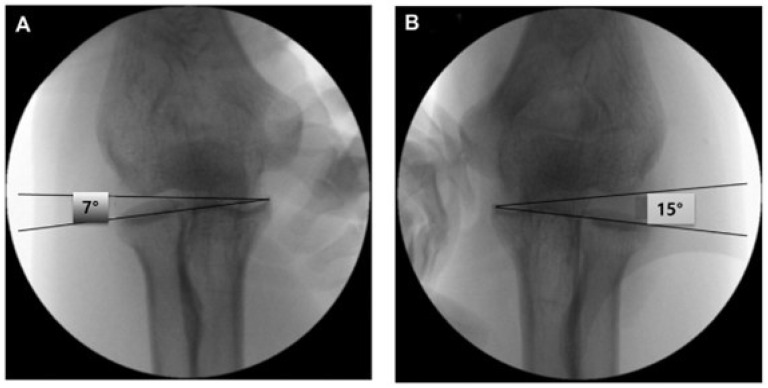
The capitellum–sublime tubercle–radial head angle (CSRA) in the normal right (**A**) and affected left (**B**) elbows of a 51-year-old male patient with recalcitrant lateral epicondylitis. The CSRA measurement reflects subtle changes in joint alignment, aiding diagnosis of minor instability in this population.

All patients underwent a standardized preoperative evaluation, including physical examination, plain radiography, and magnetic resonance imaging (MRI). Patients were classified into the unstable group (mild elbow instability) if they met the following criteria:

1. Lateral joint line tenderness and pain during the varus stress test on physical examination. This apparent discrepancy with normal CSRA in some cases reflects the possibility of symptomatic minor instability detectable only under anesthesia or dynamic testing.
2. Increased T2-weighted signal intensity of the lateral ligament complex on MRI.
3. Positive intraoperative fluoroscopic varus stress test under anesthesia.
4. A capitellum–sublime tubercle–radial head angle (CSRA) greater than 5° compared with the contralateral side. (Figure 2) This threshold was adopted based on previous studies that demonstrated its diagnostic utility in detecting subtle lateral elbow instability [13]. The CSRA measurement reflects subtle changes in joint alignment, aiding diagnosis of minor instability in this population.

Patients who did not meet these criteria were classified into the stable group. All abbreviations used in the figures are defined in the figure legends for clarity.

MRI assessments were independently reviewed by two musculoskeletal radiologists who were blinded to patient groupings. Inter-rater reliability for CSRA measurements between two musculoskeletal radiologists was calculated, yielding an ICC of 0.92.

### 1.3. Operative Technique

- Unstable Group

All procedures were performed by a single senior orthopedic surgeon with subspecialty training in elbow surgery. After general anesthesia, intraoperative stress testing with fluoroscopy was performed to confirm RCL insufficiency.

**Figure 3:**
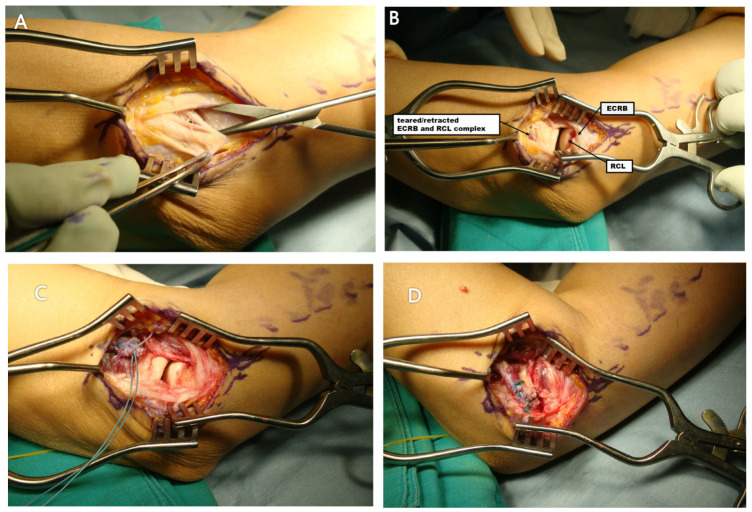
Operative technique for the unstable group: The extensor carpi radialis brevis (ECRB) tendon was found detached from its insertion between the extensor carpi radialis longus (ECRL) and extensor digitorum communis (EDC) (**A**). Following retraction of the ECRL and EDC, both the retracted ECRB and a torn radial collateral ligament (RCL) were clearly exposed (**B**). These structures were reattached using the Krackow suture technique (**C**), and a postoperative view demonstrated the repaired ECRB secured with a suture anchor (**D**). Abbreviations: ECRB, extensor carpi radialis brevis; ECRL, extensor carpi radialis longus; EDC, extensor digitorum communis; RCL, radial collateral ligament.

A curvilinear lateral incision was made over the lateral epicondyle. After fascial dissection, the common extensor origin and lateral ligament complex were exposed by retracting the extensor carpi radialis longus and extensor digitorum communis. Degenerative tissue and partial tears of the ECRB and RCL were debrided. A single suture anchor (BioCompositeSutureTak, 3.0 mm; Naples, FL, USA) was used to reattach the RCL and ECRB to the lateral epicondyle using the Krackow technique with the elbow in slight flexion and valgus. Additional horizontal mattress sutures were placed to reinforce the repair. In cases with intraoperative evidence of LUCL injury, internal brace augmentation was performed using FiberTape and SwiveLock anchors (3.5 and 4.75 mm, Arthrex, Naples, FL, USA) (Figure 3).

- Stable group

Patients in the stable group underwent the routine arthroscopic debridement of the ECRB origin and removal of degenerative tissue without any ligament repair.

### 1.4. Postoperative Rehabilitation

After the operation, the elbow was placed in a long-arm splint with the joint maintained at 90° flexion for the first week. This was followed by application of a hinged brace, which continued to hold the elbow at 90° while allowing a gradual increase in motion. Passive range-of-motion (ROM) exercises were initiated at 2 weeks, progressing to active ROM at 6 weeks. Strengthening exercises were introduced at 8 weeks, and patients were allowed to resume full activities approximately 3 months after surgery.

### 1.5. Clinical Outcome Measures

Preoperative and postoperative evaluations included:

- Visual Analog Scale (VAS) for pain.
- Mayo Elbow Performance Score (MEPS).
- Disabilities of the Arm, Shoulder and Hand (DASH) score.
- Range of motion (ROM) in flexion and extension.

Assessments were performed by a blinded clinician not involved in the surgery. Clinical follow-ups were scheduled at 6 weeks, 3 months, 6 months, and 12 months postoperatively.

### 1.6. Statistical Analysis

The subgroup comparison between the stable and unstable groups was planned a priori. No formal power analysis was performed due to the retrospective nature and limited sample size. All statistical analyses were performed using SPSS version 18.0 (SPSS Inc., Chicago, IL, USA). The normality of data distribution was assessed using the Shapiro–Wilk test. Continuous variables were compared using independent *t*-tests or Mann–Whitney U tests as appropriate. Categorical variables were analyzed using chi-square or Fisher’s exact tests. Paired comparisons (pre- vs. postoperative) were evaluated with the Wilcoxon signed-rank test. A *p*-value < 0.05 was considered statistically significant.

## 2. Results

### 2.1. Patient Demographics

A total of 92 patients underwent surgery for recalcitrant lateral epicondylitis between 2016 and 2022. Of these, 12 patients (5 men and 7 women; mean age: 54 ± 1.8 years) were classified as having mild lateral elbow instability based on the defined criteria and underwent additional ligament repair. The remaining 80 patients (31 men and 49 women; mean age: 51 ± 0.7 years) were assigned to the stable group. All 12 patients in the unstable group had a history of corticosteroid injection at the lateral epicondyle, with an average of 3.4 ± 0.9 injections. In comparison, the stable group had received fewer injections (mean 1.2 ± 0.7), and none had undergone prior ligament repair. The difference in the number of prior injections between groups was statistically significant (*p* = 0.024). Three patients (25%) in the unstable group had undergone previous surgical treatment (ECRB debridement) at outside institutions; two via arthroscopic approach and one via open technique. All three were re-operated due to persistent symptoms and had no prior documentation of elbow instability (Table 1). The mean of 3.4 corticosteroid injections was administered over an average of 18 months.

### 2.2. Trauma and Functional Impairment

Two patients in the unstable group reported trauma (e.g., fall or sports injury) after their most recent steroid injection, resulting in symptom aggravation. All unstable patients reported lateral elbow pain during varus stress testing and resisted third-finger extension. Preoperative imaging and intraoperative assessments confirmed RCL insufficiency in all 12 unstable cases. The mean Capitellum–Sublime tubercle–Radial head Angle (CSRA) difference between affected and unaffected elbows exceeded 5°, consistent with mild instability (mean ΔCSRA = 6.7° ± 1.2°). Postoperative imaging showed normalized alignment in all patients. (For consistency, the term ‘mild instability’ was defined and used consistently with ‘unstable’ throughout the abstract).

### 2.3. Clinical Outcomes

Pre- and postoperative clinical outomes in unstable group were as follows: mean VAS for pain improved from 6.8 ± 1.5 to 1.4 ± 0.5 at final follow-up (*p* < 0.001). MEPS increased from 53.0 ± 10.1 to 91.1 ± 10.7 (*p* < 0.001), and DASH scores improved from 47.1 ± 10.2 to 13.8 ± 3.8 (*p* < 0.001). No patients demonstrated varus instability on follow-up examination or fluoroscopy at 1 year postoperatively. Range of motion was maintained or improved in all patients. Flexion improved from 138° ± 9.1 to 143° ± 8.1 (*p* = 0.072), and extension remained within functional range (0.3° ± 3.2 preop vs. 1.2° ± 1.5 postop, *p* = 0.413). Table 2 presents preoperative and postoperative scores for both stable and unstable groups; comparisons are indicated where statistically significant.

The repaired ECRB tendon and RCL complex appeared intact on follow-up MRI in all patients. In the stable group, postoperative MRI was not routinely performed, as ligament repair was not undertaken.

### 2.4. Complications and Reoperation

No major complications such as infection, nerve injury, or re-rupture were observed. No patient required reoperation. Mild transient stiffness was noted in two cases and resolved with physiotherapy within three months. No evidence of residual instability was observed at the final follow-up.

## 3. Discussion

Degeneration of the ECRB tendon is widely recognized as the primary etiology of lateral elbow pain [13]. In our cohort, pain was the predominant presenting symptom, and pain relief remains the fundamental therapeutic goal in the management of lateral epicondylitis. Prior studies have reported that the majority of patients achieve substantial symptomatic improvement and can return to pre-injury activity levels after surgical treatment. Nevertheless, approximately 10–20% of individuals continue to experience persistent pain that prevents resumption of prior activity levels [14]. In their classic series, Nirschl and Pettrone [3] reported the outcomes of 88 elbows in 82 patients treated with excision and repair of the ECRB lesion; 13 patients (16%) demonstrated only fair or poor results, with persistent pain during strenuous activities or no symptomatic improvement. We hypothesize that such suboptimal outcomes may have been related to unrecognized instability that was not addressed at the time of surgery. This type of pathology may develop from repetitive, forceful forearm extension combined with rotational movement, particularly when accompanied by sustained gripping [15]. Repeated mechanical loading may exceed the tendon’s physiological tolerance. Once fiber elongation surpasses the allowable threshold, the resulting internal stress can exceed the ultimate tensile capacity of the tendon, leading to structural failure. Dzugan et al. documented acute radial ulnohumeral ligament injuries in patients with chronic lateral epicondylitis, usually following traumatic events, while Kalainov and Cohen described three atraumatic cases in which clinical findings were consistent with posterolateral rotatory instability (PLRI) of the elbow [15,16].

The role of the RCL in lateral epicondylitis has not been extensively investigated. More recently, however, several reports have emphasized the significance of the RCL, as well as the synergistic contribution of the lateral ligament complex, in the pathogenesis of PLRI [17]. In an in vivo study, Moritomo et al. demonstrated that the RCL maintains near-isometric behavior throughout elbow motion and may play a more critical stabilizing role than the LUCL [18]. In our series, no patient exhibited clinical PLRI, supporting the notion that RCL pathology rather than LUCL deficiency underlies instability in recalcitrant lateral epicondylitis.

In our study, all patients reported pain during the varus stress test, although no radiographic evidence of instability was observed on static imaging (varus and valgus stress tests). Arrigoni et al. identified the RCL as an essential static stabilizer against lateral elbow instability [19]. Cadaveric research has similarly shown that repeated varus loading gradually weakens the periarticular structures surrounding the radial head. More recently, a high prevalence of intra-articular abnormalities attributed to pathological laxity of the RCL in patients with chronic lateral elbow pain has led to the proposal of the concept of SMILE (symptomatic minor instability of the lateral elbow) [20]. In our series, the diagnostic framework for mild instability was consistent with the SMILE concept, with the additional incorporation of quantitative CSRA measurements to provide greater objectivity. The presence of normal CSRA values in some cases underscores that minor instability may only be detectable under anesthesia or through dynamic stress testing.

All patients in our study had received prior corticosteroid injections. Degan et al. and Shim et al. reported that instability developed after an average of 4.6 injections in patients with recalcitrant lateral epicondylitis [17,21]. Because the lateral epicondyle is covered by relatively thin soft tissue and dense tendon–ligament structures, there is a substantial risk of inadvertent intra-ligamentous or intra-tendinous infiltration during injection. In our cohort, instability after repeated corticosteroid injections was present in all cases of the unstable group, with a mean of 3.4 injections administered over 18 months. Corticosteroids are known to suppress cellular pathways involved in tendon repair and to alter cellular differentiation [22]. Experimental studies have shown that even a single corticosteroid injection can transiently impair tendon tensile strength, although biomechanical properties generally recover over time [23,24]. In an animal model, Mikolyzk et al. demonstrated that corticosteroid exposure reduced the load to failure, stress, and stiffness of normal rotator cuff tendons by 27%, 25%, and 32%, respectively, at one week [25]. Histological evaluation revealed an increased proportion of adipocytes in steroid-exposed tendons compared with controls [26]. Repeated corticosteroid injections may therefore promote tendon degeneration and impair tendon cell viability. Degeneration of the common extensor tendon, combined with repetitive mechanical stress after steroid administration, may predispose to rupture of both tendon and adjacent ligamentous structures [17,27].

Iatrogenic injury during surgical procedures for lateral epicondylitis has also been implicated in the development of secondary instability. Morrey reported 13 cases of failed surgical treatment for lateral epicondylitis, in which three patients developed instability as a consequence of surgery [28]. In contrast, most series of arthroscopic procedures have not reported postoperative instability. In our cohort, three patients had a history of prior surgery, but no definitive correlation between surgical history and instability was observed. It is plausible that instability arises from a combination of factors, including iatrogenic LCL injury and repeated corticosteroid exposure. In such cases, instability-related pain may contribute to resistance to conservative treatment. Consistent with this notion, in our study the stable group underwent arthroscopic ECRB release and drilling, whereas the unstable group required open debridement with RCL repair.

The present study has several limitations. First, it was a retrospective case series, inherently subject to potential bias. Second, the sample size was small and heterogeneous, limiting statistical power. Third, potential confounding factors—including differences in age, number of steroid injections, and surgical history—may have influenced outcomes. In addition, the classification of stable versus unstable groups relied partly on the operating surgeon’s interpretation of physical examination and imaging findings, introducing an element of subjectivity. Future research should seek to define biomechanical thresholds by which repeated corticosteroid exposure produces functional ligament insufficiency. Prospective correlation between advanced imaging modalities and intraoperative findings may also facilitate earlier recognition of SMILE in patients with lateral elbow pathology. Furthermore, the development of a standardized clinical scoring system incorporating MRI, stress testing, and injection history could provide a more objective framework for stratifying patients and guiding surgical decision-making.

## 4. Conclusions

Favorable clinical outcomes were defined as improvements compared to preoperative baseline scores. Recalcitrant lateral epicondylitis cases should be carefully checked to see if there is an accompanying instability, in which case repair of the radial collateral ligament should be considered. Simultaneous surgical treatment with open debridement and ligament repair provides satisfactory pain relief and functional impairment in refractory lateral epicondylitis with radial collateral ligament injury, especially with multiple steroid injection history or previous surgeries on the lateral epicondyle area.

## Figures and Tables

**Table 1:** Demographics of unstable group vs. stable group.

	Unstable Group	Stable Group	*p*-Value
Sex, men/women	5/7	31/49	0.135
Age (mean ± SD), yr	54 ± 1.8	51 ± 0.7	0.042 *
Involvement of dominant arm, *n* (%)	8 (66)	44 (55)	0.908
Case with steroid injection history, *n* (%)	12 (100)	62 (78)	0.306
Case with previous surgery, *n* (%)	3 (25)	0 (0)	0.037 *
No. of steroid injections, mean ± SD	3.4 ± 0.9	1.2 ± 0.7	0.024 *
Case with trauma history, *n* (%)	2 (17)	3 (3)	0.097

* indicates statistical significance after Bonferroni correction (*p* < 0.05).

**Table 2:** Preoperative and postoperative scores for both stable and unstable groups; comparisons are indicated where statistically significant.

	**Preoperative**	**Postoperative**	***p*-Value**
pVAS	6.8 ± 1.5	1.4 ± 0.5	0.001 *
ROM			
Flexion	138 ± 9.1	143 ± 8.1	0.072
Extension	0.3 ± 3.2	1.2 ± 1.5	0.413
DASH score	47.1 ± 10.2	13.8 ± 3.8	0.001 *
MEPS			
Total score	53.0 ± 10.1	91.1 ± 10.7	0.001 *
Stability	3.2 ± 1.1	10.0 ± 1.5	0.001 *

* indicates statistical significance after Bonferroni correction (*p* < 0.05).

## Data Availability

All relevant data are included within the manuscript. No new datasets were generated or analyzed during the current study.

## References

[1] Park H.B., Kam M., Gwark J.Y. (2019). Association of steroid injection with soft-tissue calcification in lateral epicondylitis. J. Shoulder Elb. Surg..

[2] Cho B.-K., Kim Y.-M., Kim D.-S., Choi E.-S., Shon H.-C., Park K.-J., Lee E.-M. (2009). Mini-open muscle resection procedure under local anesthesia for lateral and medial epicondylitis. Clin. Orthop. Surg..

[3] Nirschl R.P., Pettrone F.A. (1979). Tennis elbow. The surgical treatment of lateral epicondylitis. J. Bone Jt. Surg..

[4] Mi B., Liu G., Zhou W., Lv H., Liu Y., Wu Q., Liu J. (2017). Platelet rich plasma versus steroid on lateral epicondylitis: Meta-analysis of randomized clinical trials. Physician Sportsmed..

[5] Arirachakaran A., Sukthuayat A., Sisayanarane T., Laoratanavoraphong S., Kanchanatawan W., Kongtharvonskul J. (2016). Platelet-rich plasma versus autologous blood versus steroid injection in lateral epicondylitis: Systematic review and network meta-analysis. J. Orthop. Traumatol..

[6] Smidt N., van der Windt D.A., Assendelft W.J., Deville W.L., Korthals-de Bos I.B., Bouter L.M. (2002). Corticosteroid injections, physiotherapy, or a wait-and-see policy for lateral epicondylitis: A randomised controlled trial. Lancet.

[7] Takigawa N., Ryu J., Kish V.L., Kinoshita M., Abe M. (2005). Functional anatomy of the lateral collateral ligament complex of the elbow: Morphology and strain. J. Hand Surg..

[8] Park C.H., Kim B.S., Lee J.H., Chung S.G. (2019). Optimal Elbow Positions for Identification of the Radial Collateral Ligament Using Ultrasonography. PM R.

[9] Cohen M.S. (2008). Lateral collateral ligament instability of the elbow. Hand Clin..

[10] Zoner C.S., Buck F.M., Cardoso F.N., Gheno R., Trudell D.J., Randall T.D., Resnick D. (2010). Detailed MRI-anatomic study of the lateral epicondyle of the elbow and its tendinous and ligamentous attachments in cadavers. Am. J. Roentgenol..

[11] Park J.Y., Bang J.Y. (2016). Spontaneous Rupture of the Extensor Carpi Radialis Brevis and Radial Collateral Ligament of the Elbow in a Recreational Golfer: Surgical Experience of Repair of a Chronic Retracted Tendon and Ligament. Clin. Shoulder Elb..

[12] Noh Y.M., Kong G.M., Moon S.W., Jang H.S., Kim S., Bak G.G., Kim Y. (2021). Lateral ulnar collateral ligament (LUCL) reconstruction for the treatment of recalcitrant lateral epicondylitis of the elbow: A comparison with open debridement of the extensor origin. JSES Int..

[13] Morrey B.F., An K.N. (1985). Functional anatomy of the ligaments of the elbow. Clin. Orthop. Relat. Res..

[14] Solheim E., Hegna J., Øyen J., Inderhaug E. (2013). Arthroscopic versus open surgical release for lateral epicondylitis: 5-year results of a randomized controlled trial. J. Shoulder Elb. Surg..

[15] Dzugan S.S., Savoie F.H., Field L.D., O’Brien M.J., You Z. (2012). Acute radial ulno-humeral ligament injury in patients with chronic lateral epicondylitis: An observational report. J. Shoulder Elb. Surg..

[16] Kalainov D.M., Cohen M.S. (2005). Posterolateral rotatory instability of the elbow in association with lateral epicondylitis. A report of three cases. J. Bone Jt. Surg..

[17] Shim J.W., Yoo S.H., Park M.J. (2018). Surgical management of lateral epicondylitis combined with ligament insufficiency. J. Shoulder Elb. Surg..

[18] Moritomo H., Murase T., Arimitsu S., Oka K., Yoshikawa H., Sugamoto K. (2007). The in vivo isometric point of the lateral ligament of the elbow. J. Bone Jt. Surg..

[19] Arrigoni P., Cucchi D., Luceri F., Menon A., Zaolino C., Zagarella A., Catapano M., Radici M., Migliaccio N., Polli D. (2021). Lateral Elbow Laxity Is Affected by the Integrity of the Radial Band of the Lateral Collateral Ligament Complex: A Cadaveric Model With Sequential Releases and Varus Stress Simulating Everyday Activities. Am. J. Sports Med..

[20] Arrigoni P., Cucchi D., D’aMbrosi R., Butt U., Safran M.R., Denard P., Randelli P. (2017). Intra-articular findings in symptomatic minor instability of the lateral elbow (SMILE). Knee Surg. Sports Traumatol. Arthrosc..

[21] Degen R.M., Cancienne J.M., Camp C.L., Altchek D.W., Dines J.S., Werner B.C. (2017). Three or more preoperative injections is the most significant risk factor for revision surgery after operative treatment of lateral epicondylitis: An analysis of 3863 patients. J. Shoulder Elb. Surg..

[22] Tempfer H., Gehwolf R., Lehner C., Wagner A., Mtsariashvili M., Bauer H.-C., Resch H., Tauber M. (2009). Effects of crystalline glucocorticoid triamcinolone acetonide on cultered human supraspinatus tendon cells. Acta Orthop..

[23] Hugate R., Pennypacker J., Saunders M., Juliano P. (2004). The effects of intratendinous and retrocalcaneal intrabursal injections of corticosteroid on the biomechanical properties of rabbit Achilles tendons. J. Bone Jt. Surg..

[24] Wiggins M.E., Fadale P.D., Barrach H., Ehrlich M.G., Walsh W.R. (1994). Healing characteristics of a type I collagenous structure treated with corticosteroids. Am. J. Sports Med..

[25] Mikolyzk D.K., Wei A.S., Tonino P., Marra G., Williams D.A., Himes R.D., Wezeman F.H., Callaci J.J. (2009). Effect of corticosteroids on the biomechanical strength of rat rotator cuff tendon. J. Bone Jt. Surg..

[26] Tillander B., Franzen L.E., Karlsson M.H., Norlin R. (1999). Effect of steroid injections on the rotator cuff: An experimental study in rats. J. Shoulder Elb. Surg..

[27] Arslan I., Yucel I., Ozturk T.B., Karahan N., Orak M.M., Midi A. (2019). The Effects of Corticosteroid Injection in the Healthy and Damaged Achilles Tendon Model: Histopathological and Biomechanical Experimental Study in Rats. Turk. J. Pathol..

[28] Morrey B.F. (1992). Reoperation for failed surgical treatment of refractory lateral epicondylitis. J. Shoulder Elb. Surg..

